# Metaphyseal anchoring short stem hip arthroplasty provides a more physiological load transfer: a comparative finite element analysis study

**DOI:** 10.1186/s13018-020-02027-4

**Published:** 2020-10-29

**Authors:** Shuang G. Yan, Yan Chevalier, Fanxiao Liu, Xingyi Hua, Anna Schreiner, Volkmar Jansson, Florian Schmidutz

**Affiliations:** 1 Department of Orthopaedic Surgery, The First Affiliated Hospital of Anhui Medical University, No.1 Baicao Road, Hefei, 230088 China; 2 Department of Orthopaedic Surgery, Physical Medicine and Rehabilitation, University of Munich (LMU), Munich, Germany; 3 Department of Orthopaedics, Shandong Provincial Hospital affiliated to Shandong First Medical University, No. 324, Road Jing Wu Wei Qi, Jinan, 250021 Shandong China; 4 BG Trauma Center Tuebingen, Eberhard Karls University Tuebingen, Schnarrenbergstrasse 95, 72076 Tuebingen, Germany

**Keywords:** Stress shielding, Finite element analysis, Total hip arthroplasty, SHA, Stem

## Abstract

**Background:**

Short stem total hip arthroplasty (SHA) preserves femoral bone stock and is supposed to provide a more natural load transfer compared to standard stem total hip arthroplasty (THA). As comparative biomechanical reference data are rare we used a finite element analysis (FEA) approach to compare cortical load transfer after implantations of a metaphyseal anchoring short and standard stem in native biomechanical femora.

**Methods:**

The subject specific finite element models of biomechanical femora, one native and two with implanted metaphyseal anchoring SHA (Metha, B. Braun Aesculap) and standard THA (CLS, Zimmer-Biomet), were generated from computed tomography datasets. The loading configuration was performed with an axial force of 1400 N. Von Mises stress was used to investigate the change of cortical stress distribution.

**Results:**

Compared to the native femur, a considerable reduction of cortical stress was recorded after implantation of SHA and standard THA. The SHA showed less reduction proximally with a significant higher metaphyseal cortical stress compared to standard THA. Moreover, the highest peak stresses were observed metaphyseal for the SHA stem while for the standard THA high stress pattern was observed more distally.

**Conclusions:**

Both, short and standard THA, cause unloading of the proximal femur. However, the metaphyseal anchoring SHA features a clearly favorable pattern in terms of a lower reduction proximally and improved metaphyseal loading, while standard THA shows a higher proximal unloading and more distal load transfer. These load patterns implicate a reduced stress shielding proximally for metaphyseal anchoring SHA stems and might be able to translate in a better bone preservation.

## Background

Total hip arthroplasty (THA) has become a surgical procedure with excellent results in patients with severe degenerative or traumatic arthritis of the hip [1, 2]. Cementless THA has recently become a standard procedure and is preferentially used in younger patients [3, 4]. However, despite the excellent outcome and long-term results of the cementless THA systems, failure of the implants still occurs [5]. Failure and loosening of THA is often characterized by bone loss [6] and compromises revision and anchorage of further implants. Thus, conservation of bone stock is an important principle, particularly in young patients.

Stress shielding, referring to the reduction of load transferred to the surrounding bone, is an important factor to cause bone resorption and implant failure [7]. Different variables, but especially stem geometry and design are key factors for the load transfer and bone remodeling at the femur [8, 9]. Especially straight standard THA stems are prone to cause stress shielding with a proximal unloading and more distal load transfer. Lately, short stem hip arthroplasty has been introduced, and besides a shorter femoral stem the results from biomechanical experiments indicate a better stress distribution with an improved loading of the femur [10, 11]. However, most of these studies used a biomechanical setup comparing primary stability and strain distribution between short stem total hip arthroplasty (SHA) and THA [10]. Nevertheless, it is well known for SHA that obvious differences exist in the load transfer between the various stem designs which are mainly related to their differing anchoring concepts [12]. Clinical DXA data evaluating the bone remodelling showed a more balanced load transfer for predominantly proximal or metaphyseal anchoring SHA implants compared to more distal anchoring SHAs, showing the need to assess them separately [12].

Finite element analysis (FEA) is a standard tool used in biomedical engineering to precisely assess stress distribution in a wide range of femoral geometries, once material properties, loading and boundary conditions have carefully been selected. Several FEA studies assessed the stress distribution around THA stems [13–15] and also were used to simulate adaptive bone density remodeling [16].

To our knowledge, no FEA study directly compared the cortical stress in the proximal femur after implantations of a cementless metaphyseal anchoring SHA and THA. Therefore, the present study evaluated the effect of metaphyseal anchoring SHA and THA on cortical stress shielding. According to the current literature, we hypothesized that a SHA stem is able to restore the load transfer more physiologically compared to a standard THA stem.

## Materials and methods

### Implants

**Fig. 1:**
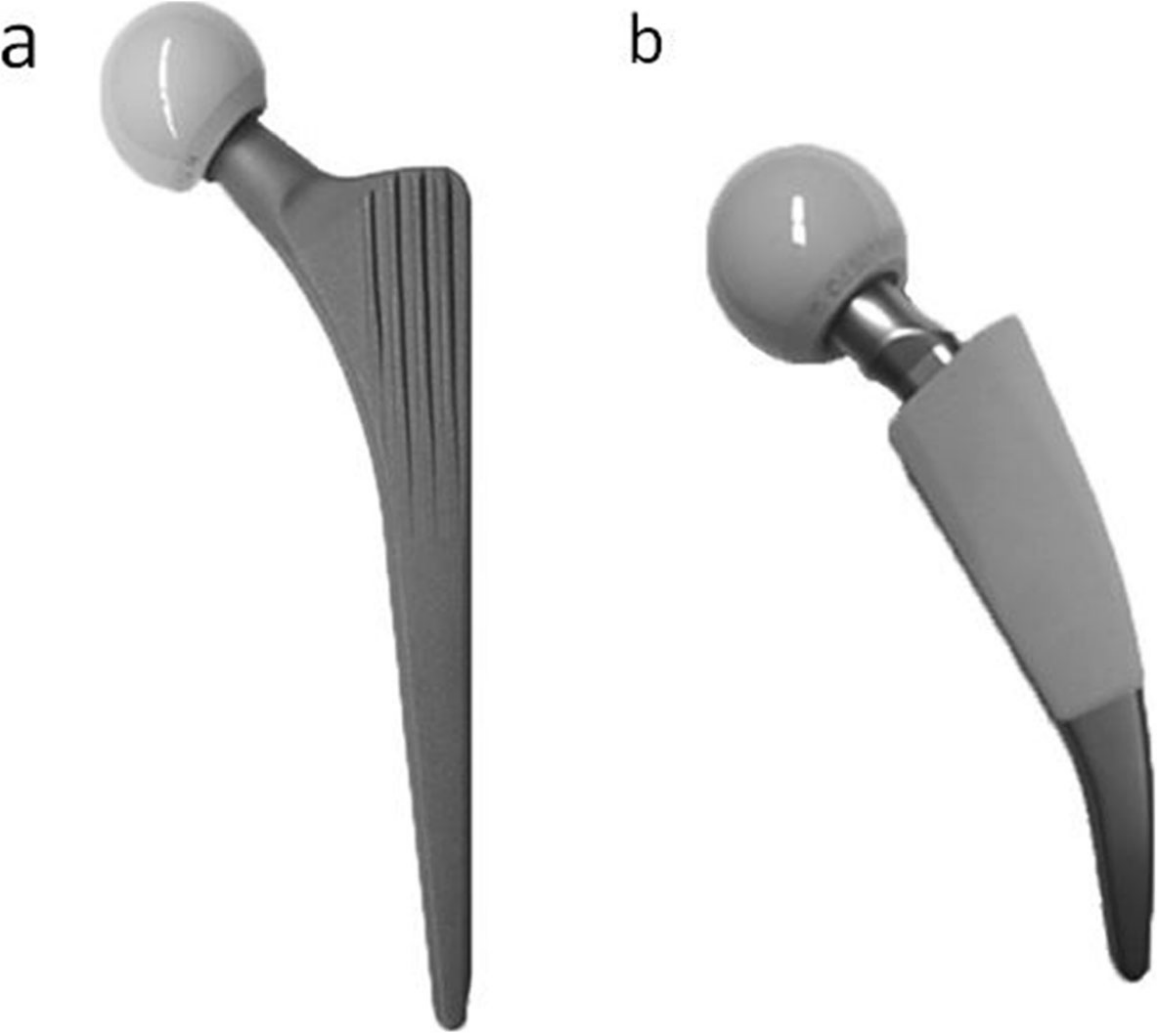
Femoral stems. **a** Cementless metaphyseal anchoring short stem (SHA: Metha Aesculap, B. Braun) and **b** cementless standard straight stem (THA: CLS, Zimmer-Biomet)

The Metha short stem (B. Braun, Aesculap, Tuttlingen, Germany) (Fig. 1a) is a cementless partial collum sparing implant with a proven metaphyseal anchorage [17, 18]. It has a 20-μm-thick Calciumphosphate layer in the proximal and middle part, and is polished distally. According to previous studies, a Metha stem size 3 with a 135° adapter was implanted [19, 20].

The CLS standard stem (Zimmer, Warsaw, IN, USA) (Fig. 1b) is a cementless, straight, and collarless implant with a proximal anchorage and proven good long-term results. It has a porous surface treatment (Ra = 4.4 μm) and a rectangular cross-section with sharp proximal, anterior and posterior ribs. According to previous studies, a CLS prostheses size 13.25 with a 135° neck-shaft-angle was used [21, 22].

### Specimen preparation

In order to acquire the FE models, the specimens were derived from ongoing biomechanical experiments evaluating the micromotions in our laboratory [20] (Fig. 1). This study used synthetic composite bones (Model 3306, sawbones Pacific Research Laboratories, USA) to avoid geometric and mechanical variances as seen in cadaveric bones [23]. Besides, the composite bones were found to have analog bone properties and mimic the structural properties of average healthy adult human bones [24].

The femora are positioned laterally by 9° in the frontal plane and dorsally by 16° in the sagittal plane to create physiological loading conditions [25]. All implantations were performed by one senior surgeon (FS) according to the manufacturers’ instructions. A sinusoid dynamic load was applied downward vertically with an amplitude between 300 N to 1700 N and a frequency of 1 Hz, to simulate a post-operative patient with 70 kg body weight walking on level ground [25].

### FE models

**Fig. 2:**
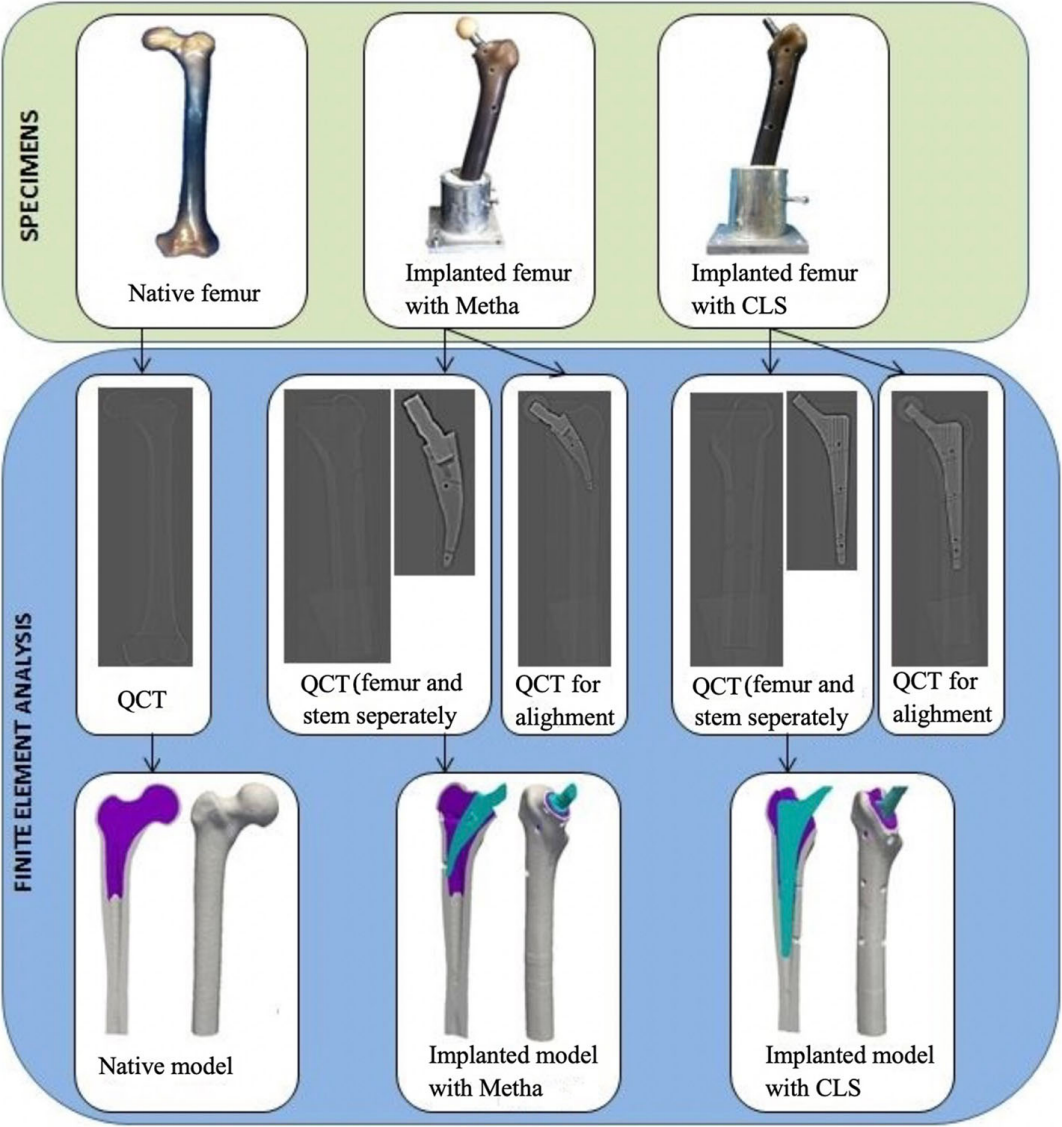
Workflow for the generations of the homogenized FE models

One intact native composite femur and two composite femora, one with implanted SHA (Metha) and one with THA (CLS), were used to create the FE models. The homogenized FE models were generated as outlined in Fig. 2. Details about the model generation are provided in the following subsections in accordance to the four modeling steps: (1) three-dimensional (3D) model generation of the femurs and implants in the clinical quantitative computerized tomography (QCT) scans of the prepared samples, (2) alignment of the implants, (3) material properties assignment, and (4) boundary condition assignment. The final subsection dealt with model solving and post-processing. Except stated otherwise, all these steps were conducted using custom-made programs in Python, C++ and Fortran as recently described by Chevalier Y [26]..

### QCT scanning and 3D model generation

QCT scans of the implanted and intact specimens, as well as of the two selected femoral stems were conducted using a clinical computed tomography (CT) scanner (64-slice) (Siemens Somatom Emotion 6, Siemens AG, Germany). 3D images were reconstructed with a voxel size of approximately 0.17 × 0.17 × 0.6 mm^3^. Trabecular and cortical bone were segmented from the CT scans based on gray-scale transition values using in-house written code [22]. Furthermore, 3D models of the two prostheses were also created after segmentation of the implant in the implant scan images.

### Alignment of the implants

To assure the accurate implant position, the 3D models of the isolated SHA and THA stems were placed by aligning them to the positions as recorded in the CT scans of the femur with the implanted stem. The positioned implant models were then converted to digitized images with custom codes in Python and insight toolkit (ITK) [22]. Then, the bone and implant images were combined into a binarized image with three distinct regions (compact bone, trabecular bone, and stem), and then meshed with 2-mm 4-noded tetrahedral with computational geometry algorithms library (CGAL) [27] to create 3D models of the femurs with stems as described previously [22]. The merged models of SHA (Metha) and THA (CLS) femur contained between 52 and 58 × 10^3^ nodes, and 21 and 24 × 10^4^ elements, respectively. The merged model of the native femur contained approximately 66 × 10^3^ nodes and 29 × 10^4^ elements.

### Material properties assignment

Synthetic composite femur was characterized by isotropic material properties of cortical bone and trabecular bone, which were assumed to be linearly elastic and homogeneous with Poisson’s ratio setting to 0.35. Cancellous stiffness modulus value was designed by 155 MPa, and cortical stiffness value was designed by 16.7 GPa. The stiffness modulus values of the SHA and THA stem were designed by 25 GPa.

### Boundary condition assignment

Models were loaded to mimic the experimental conditions of the specimens as in the in vitro study [20]. Loading vector was defined based on the anatomical orientation and corresponded to a 9° angle in the frontal plane and 16° angle in the sagittal plane. A resultant load with 1400 N was applied on the tip nodes of the prosthesis neck, while bottom nodes of the bone were fully constrained.

### Solving and post-processing

Linear analyses were performed using Abaqus 6.13 (Simulia, Dassault Systèmes, Vélizy-Villacoublay, France). To analyze the cortical stress distribution patterns of the FE models, a custom code was written dividing the FE models into equal regions of 10 mm starting from proximal to distal in the *z*-axis direction. The mean and peak values of von Mises stress in each region were then computed. To further analyze the mean cortical stress distribution around the stems, the femora were divided into a proximal (region 1-6), metaphyseal (region 7–12) and distal (region 13–18) region. Visualization of the mean stresses for the FE models was done in Paraview v3.14.

### Statistics

The FEA results (Native, SHA, and THA) are depicted and described comparatively for the mean cortical stress distributions and peak cortical stress distributions. To further analyze the mean cortical stress distributions in the proximal, metaphyseal, and distal region of the three groups, one-way analysis of variance (with a Bonferroni post hoc test) was conducted. Data analysis and graphic representation were conducted using GraphPad Prism 5 (GraphPad Software, San Diego, USA). The level of significance was set at 0.05.

## Results

**Fig. 3:**
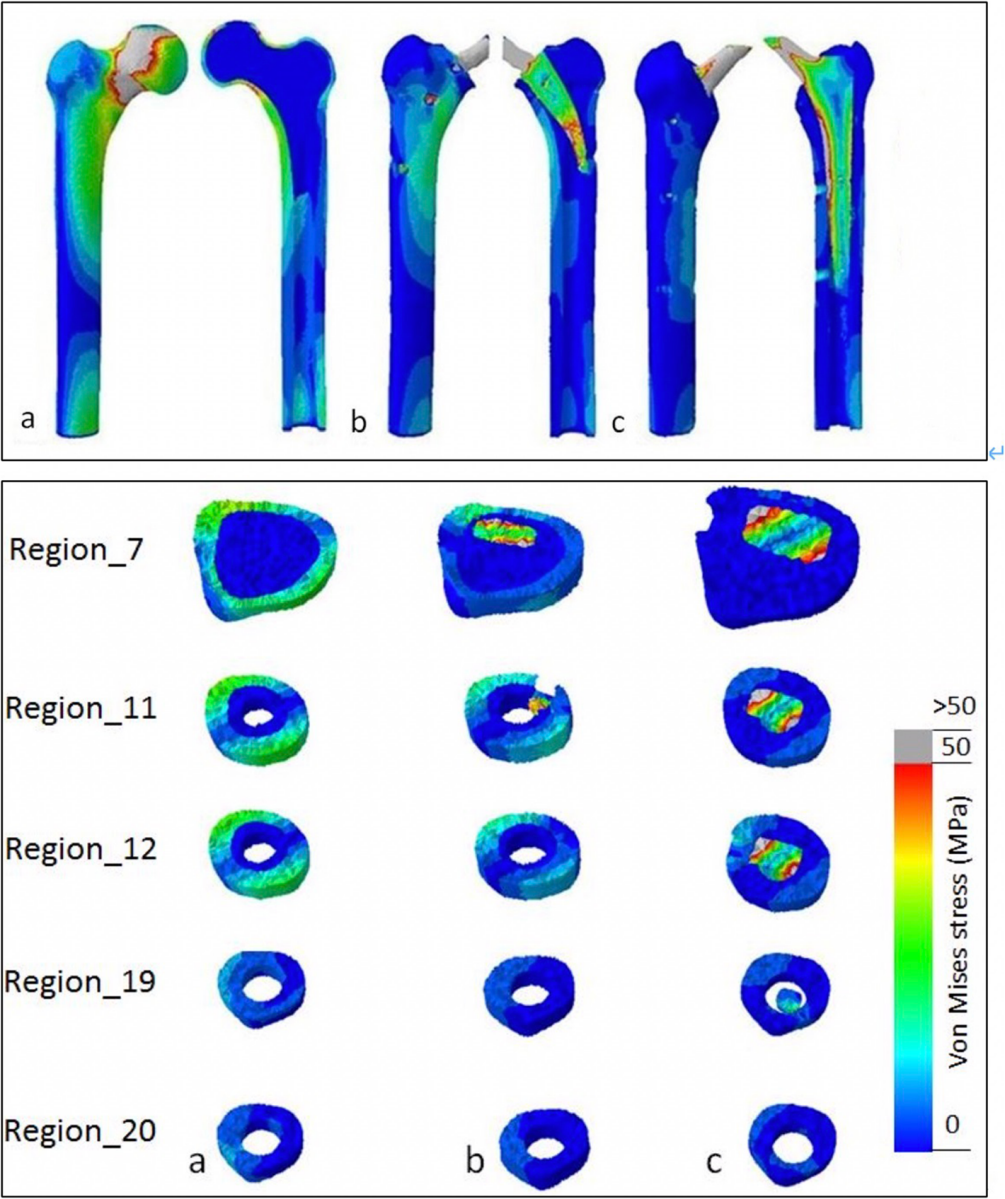
Coronal and transverse views of von Mises stress distribution patterns in the three FE models: **a** native bone, **b** short stem (SHA, Metha), and **c** standard stem (THA, CLS). Load transfer for the native bone is predominantly proximally at the cortex, while after short and standard stem insertion the load transfers in large parts via the implant. However, the short stem shows a clear metaphyseal load transfer while for the standard stem the load transfer is more distally

**Fig. 4:**
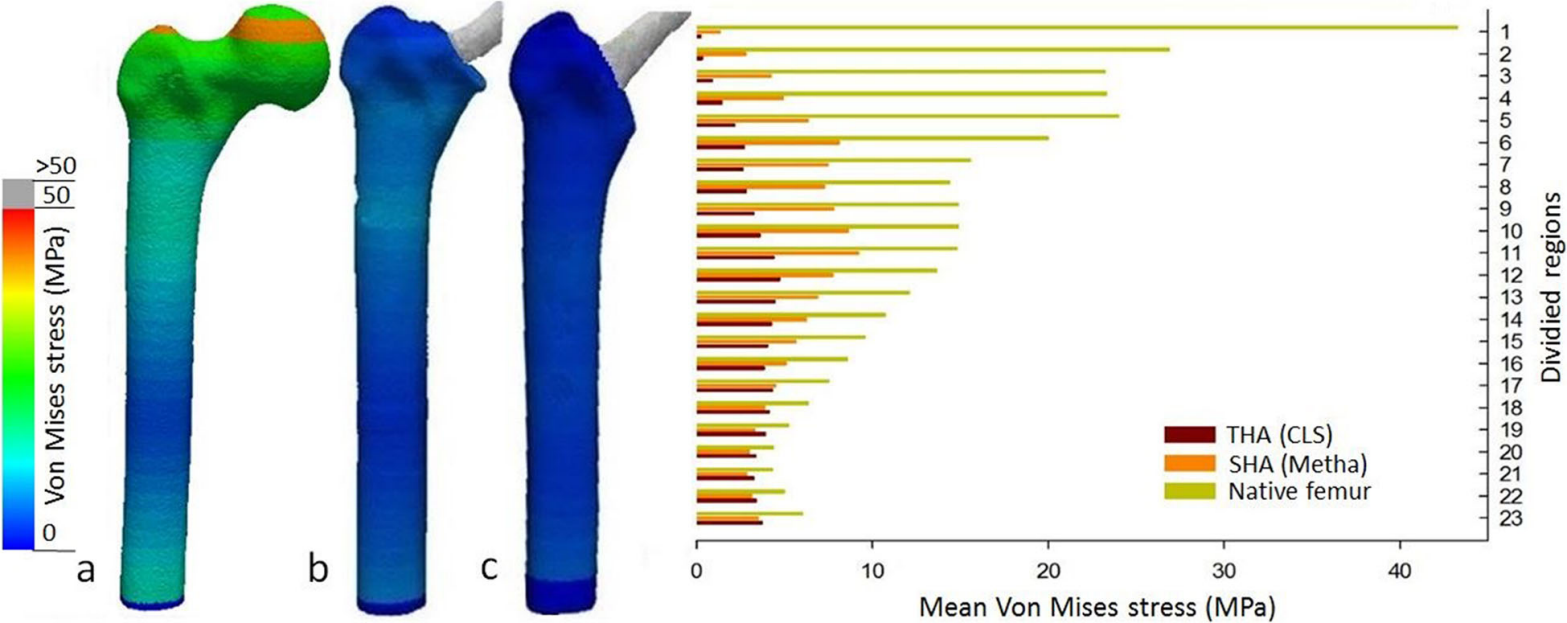
Mean cortical stress distributions in the three FE models (front view): **a** native cortical bone, bone with implanted, **b** short stem (Metha), and **c**)standard stem (CLS). Clear reduction of the cortical stress after implantation of both stems in the proximal region, however, with better metaphyseal loading of the short stem

**Fig. 5:**
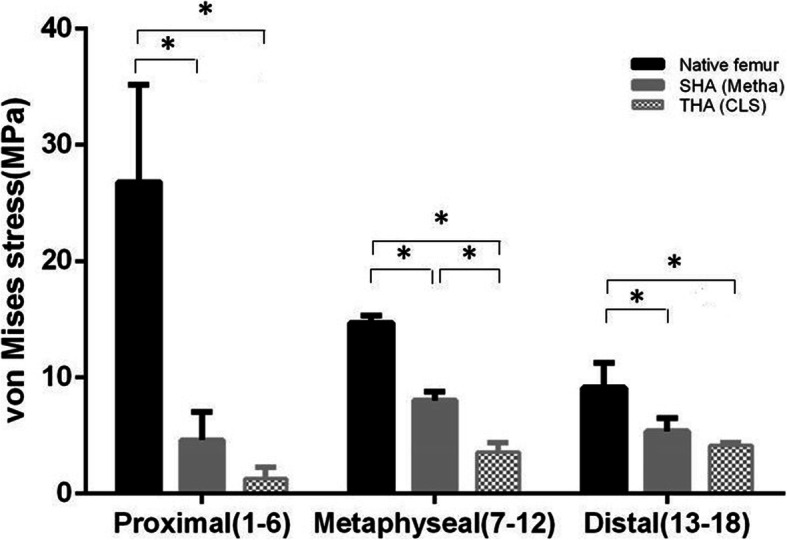
Mean cortical stress distributions for the different FE models (Native, SHA and THA) in the proximal, metaphyseal and distal femoral regions. Asterisk (*) indicates significance to the CLS-primary (*p* < 0.05). Clear reduction of the cortical stress after SHA and THA with high unloading of the proximal region. Notable, SHA shows a significantly improved loading of femur and a clearly improved metaphyseal loading compared to THA

**Fig. 6:**
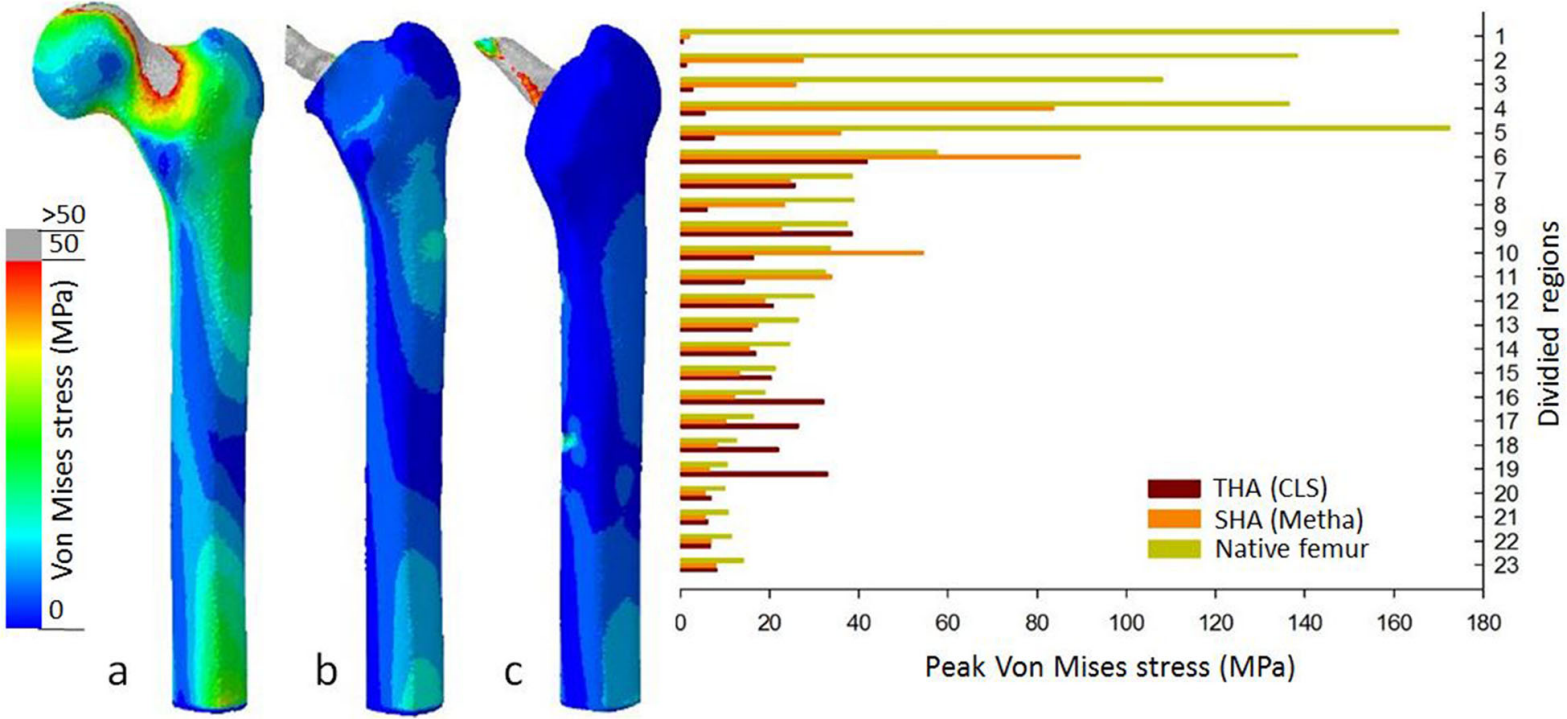
Peak cortical stress distributions in the FE models (native, SHA, and THA) (post view): **a** native bone, bone with implanted, b short stem (Metha), and **c** standard stem (CLS). Higher cortical loading for the SHA in the proximal region 2–6, while the standard stem shows high peak stress in the distal region 16–19

The results of the cortical stress distribution are shown in coronal and transverse views in Fig. 3. The mean cortical stress distribution for the different regions are given in Tables 1 and 2 and depicted in Figs. 4 and 5. Peak cortical stresses are shown in Fig. 6.

**Table 1:** Mean and peak von Mises stresses

	Mean stress (MPa)			Peak stress (MPa)		
Cortex region	Native bone	SHA (Metha)	THA (CLS)	Native bone	SHA (Metha)	THA (CLS)
1	43.26	1.33	0.25	160.84	1.97	0.60
2	26.87	2.79	0.31	138.23	27.40	1.17
3	23.23	4.23	0.88	107.96	25.79	2.69
4	23.32	4.92	1.43	136.46	83.67	5.38
5	24.00	6.31	2.16	172.44	35.74	7.42
6	19.99	8.09	2.72	57.40	89.44	41.70
7	15.56	7.45	2.62	38.37	24.40	25.66
8	14.39	7.28	2.82	38.82	23.29	5.83
9	14.90	7.77	3.24	37.38	22.44	38.39
10	14.88	8.63	3.61	33.43	54.31	16.27
11	14.77	9.19	4.38	32.31	33.77	14.30
12	13.65	7.73	4.72	29.77	18.89	20.67
13	12.08	6.89	4.45	26.29	17.10	15.93
14	10.71	6.25	4.24	24.34	15.39	16.87
15	9.58	5.64	4.01	21.14	13.15	20.28
16	8.57	5.07	3.83	18.84	12.08	32.01
17	7.49	4.49	4.31	16.20	10.09	26.37
18	6.35	3.85	4.11	12.50	8.06	21.89
19	5.23	3.30	3.90	10.35	6.47	32.89
20	4.37	2.99	3.36	9.90	5.46	6.76
21	4.30	2.85	3.24	10.68	5.52	6.04
22	4.97	3.12	3.37	11.37	6.74	6.59
23	5.98	3.47	3.71	13.98	7.90	8.00

**Table 2:** Means stresses and comparison of the different femoral zones

	Mean stress (MPa) (mean ± SD)	Comparison mean stress	***p*** value
**Proximal (1–6)**			
Native femur	26.78 ± 8.37	Native vs. SHA	< 0.0001
SHA (Metha)	4.61 ± 2.42	Native vs. THA	< 0.0001
THA (CLS)	1.29 ± 1.01	SHA vs. THA	0.8213
**Metaphyseal (7–12)**			
Native femur	14.69 ± 0.64	Native vs. SHA	< 0.0001
SHA (Metha)	8.01 ± 0.74	Native vs. THA	< 0.0001
THA (CLS)	3.56 ± 0.84	SHA vs. THA	< 0.0001
**Distal (13–18)**			
Native femur	9.13 ± 2.11	Native vs. SHA	0.0008
SHA (Metha)	5.36 ± 1.12	Native vs. THA	< 0.0001
THA (CLS)	4.16 ± 0.22	SHA vs. THA	0.4550

### Mean cortical stress

Considering the cortical stress distribution of the three FEA models (native femora, SHA, and THA), the insertion of both implants highly alters the load transfer compared to the native femora (Table 1 and Fig. 3). In the native femur the cortical stress is predominantly visible along the medial-anterior and lateral-posterior cortex as displayed for region 7, 11, and 12. The cortical load further decreases gradually from proximal to distal region 19–20 (Fig. 3).

While cortical stresses in the native femur are highest proximally, the stresses after SHA and THA are predominantly transferred via the stem (Table 1 and Fig. 3). The proximal cortical regions after insertion of SHA and THA are unloaded and thus the mean cortical stress in the proximal region 1–6 is significantly reduced (*p* < 0.0001) (Fig. 5). In the proximal region, the cortical stress in SHA was only 20% and 6% for THA compared to the native femur (Figs. 4 and 5).

In the metaphyseal region 7–12, SHA shows the main cortical stress, while the THA implant still shows a clear reduced cortical stress (Figs. 4 and 5, Table 2). Compared to the native femur the cortical load was reduced by 55% for SHA and 24% for THA. However, the cortical stress of SHA in the metaphyseal region was significantly (*p* < 0.0001) higher compared to THA (Fig. 5).

### Peak cortical stress

The differences in the load transfer of SHA and THA also translate into the peak cortical stress (Fig. 6). For the native femur, the highest peak cortical stress was observed proximally (region 1–5) and evenly decreased to distal femur. For SHA, the highest peak stresses were observed in proximal regions 4 and 6 as well as metaphyseal regions 10 and 11, documenting a shift to the metaphyseal region. In contrast, for THA the peak, cortical stress showed a more pronounced shift distally. The highest peak cortical stresses were observed not only in regions 6 and 9 but also in the very distal region 16–19. In the distal regions, the peak cortical stress even exceeded those of the native femoral bone.

## Discussion

In this FEA study, significant differences in cortical stresses were found in the proximal femur when analyzing metaphyseal anchoring short (SHA, Metha) and standard (THA, CLS) stem hip arthroplasty. Both stems induced a clearly reduced cortical stress at the proximal femur; however, SHA was able to realize better proximal and metaphyseal stress transfer compared to THA, indicating a more physiological femoral loading.

A considerable reduction of cortical stress was found in the proximal femur after SHA and THA, which is a well-known phenomenon reported by multiple studies [11, 12, 28, 29]. In the native bone load transfer occurs via the subchondral bone and is transferred distally [30]. Insertion of any implant into the femoral cavity subsequently changes this pattern and bypasses the load via the implant to the distal femoral bone [29]. This phenomenon of unloading the proximal femur by shielded it from stress is known as stress-shielding, which is prone to cause periprosthetic bone loss contributing to aseptic loosing or periprosthetic fractures [7, 31].

It is well known that the proximal femur is the most affected region [32], the reason why new stem designs aim for a more proximal loading. Short hip stems lately represent an alternative to conventional stem and although long-term studies are pending, short- and mid-term results are promising [33]. Two main advantages have emerged: the preservation of soft tissue and bone stock [34] as well as the assumption of an improved femoral load transfer [35]. Nevertheless, also within the group of SHA stems, a high variability in the design and thus load transfer has been demonstrated [12].

The results of this study strengthened this assumption as the FE analyses demonstrated that metaphyseal anchoring SHA succeeds to realize better proximal and metaphyseal load transfer. Still, the very proximal region shows less cortical stress compared to the native bone, but SHA offered clearly higher cortical stress transfer in the proximal regions compared to standard THA.

This matches well with clinical studies demonstrating that SHA can provide an improved bone remodeling compared to standard THA [9, 36]. In accordance to our FE analysis, a dual-energy X-ray absorptiometry (DXA) study by Lerch et al. found a clear reduction of bone mineral density (BMD) in the greater trochanteric region, while the metaphyseal BMD increased [36]. These results have similarly been reported by others [37–39] and are in line with our FEA results with the highest mean and peak cortical stress recorded metaphyseal. No relevant change in femoral BMD was noted in the most distal region [36] confirming our findings of a more proximal load transfer compared to THA. For standard THA, the proximal region showed clearly less cortical stress compared to SHA and the highest peak stresses were observed distally, arguing for a more distal shift of the load transfer than SHA.

These findings are confirmed by a meta-analysis of randomized controlled trials reporting on a superior bone remodeling for SHA with similar survival rates and clinical outcomes compared to THA [40]. Likewise, a systematic review of clinical DXA studies by Yan et al. compared the bone remodeling for different SHA and THA designs. Despite SHA could not completely avoid bone loss in the trochanteric region, most SHA designs showed a more balanced and reduced bone remodeling compared to standard THA [12]. The positive effect of an improve load transfer was most distinct for SHA designs featuring a metaphyseal or predominant proximal anchorage. This shows the need that SHA implants should not be judged as a single group, but rather should be evaluated individually or according to their anchorage pattern.

The clinical observations of an improved load transfer for SHA is also confirmed by in vitro studies, reporting that the stress reduction in the proximal femur was less in SHA than in standard THA [10, 11, 41]. Gronewold et al. demonstrated by measuring strain pattern in synthetic femora that SHA reached a much closer strain pattern proximally compared to standard THA [41]. Bieger et al. measured strain pattern and micromotions with a biomechanical setup and showed that the SHA stem could realize a better strain pattern proximally, but could not completely avoid stress shielding in Gruen zones 1 and 7 [10].

Other FEA studies evaluating stress and bone remodeling in SHA found similar results [30, 42, 43]. Lerch et al. described a BMD reduction for a short-stemmed femoral implant in the trochanteric region, a metaphyseal load, but no adverse effects distally which is in accordance to our results [43]. Razfar et al. evaluated the stress changes in the proximal humerus after short, stemless, and standard shoulder implants [30]. Their findings for the humerus correspond very well with ours for the hip and they concluded that stress shielding cannot completely be avoided, but may be reduced through the use of shorter implants [30].

Nevertheless, comparison with other FEA studies must be interpreted cautiously due to the diversities in the simulating approaches, bone-implant interface, loading conditions and specimens [13, 15]. Besides, frictional face-to-face contact and frictionless node-to-node contact are used to describe the bone-implant interface [13, 15], while in this study the bone-implant interface was bonded.

Further limitations have to be discussed and are closely linked to the FEA design and method applied. Firstly, the inner bone surface shared the same interface with the outer implant surface. This was different with the experimental study, the latter allowed contact between the bone and implant as well as slide and penetration, which might have an effect on the cortical stress distribution pattern after implantation. Second, there were only two specific implanted FE models, and subjected to simple loading configurations without muscle forces. Third, the material properties designed in the FEA might not exactly represent the actual properties in experimental study, because only the compressive moduli were used in the FEA, regardless of longitudinal and transverse tensile moduli. Fourth, the load transfer pattern was estimated according to the mean and peak cortical von Mises stress in divided regions, regardless of the direction of stress, such as compressive or tensile stress. Last, the stress distribution patterns were estimated by FEA without being validated experimentally. Nevertheless, the results match well with current clinical data especially remodeling of the BMD around both implants [12].

## Conclusions

This FEA study evaluated stress changes in the proximal femur after the implantation of a metaphyseal short and standard hip stem. Both stems caused clear reduction of the cortical stress in the proximal femur, however with apparent differences. While the short stem was characterized by a metaphyseal load transfer, the standard stem featured a combination of metaphyseal and more diaphyseal load transfer. Overall, the metaphyseal SHA stem showed an improved loading of the proximal femur and was able to better mimicked cortical stresses compared to the native femur. However, it has to be noticed that this only accounts for this type of metaphyseal anchoring SHA stem and not for all SHA implants in general. Long-term clinical results need to validate these effects especially in terms of the femoral bone mineral density.

## Data Availability

Data are available from the corresponding author upon reasonable request.

## Abbreviations

SHA: Short stem total hip arthroplasty
THA: Total hip arthroplasty
FEA: Finite element analysis
3D: Three-dimensional
QCT: Quantitative computerized tomography
CT: Computed tomography
ITK: Insight toolkit
CGAL: Computational geometry algorithms library
BMD: Bone mineral density
DXA: Dual-energy X-ray absorptiometry
SD: Standard deviation
